# Fifteen years of experience with frontalis suspension using polytetrafluoroethylene (Gore-Tex®) suture in blepharoptosis repair

**DOI:** 10.5935/0004-2749.20200005

**Published:** 2020

**Authors:** Fabio Henrique Luiz Leonardo, Allan C. Pieroni Gonçalves

**Affiliations:** 1 Department of Ophthalmology, Faculdade de Medicina do ABC, Santo André, SP, Brazil; 2 Division of Ophthalmology, Faculdade de Medicina, Universidade de São Paulo, São Paulo, SP, Brazil

**Keywords:** Eyelids/surgery, Blepharoptosis/surgery, Blepharoplasty/methods, Polytetrafluoroethylene/utilization, Suture, Posto perative period, Pálpebras/cirurgia, Blefaroptose/cirurgia, Blefaroplastia/métodos Politetrafluoretileno/utilização, Sutura, Período pós-operatório

## Abstract

**Purpose:**

To review the outcomes of frontalis suspension surgeries with the use of polytetrafluoroethylene in patients with blepharoptosis.

**Methods:**

A retrospective observational study analyzed the outcomes of frontalis suspension surgeries performed in a single institution from 2003 to 2018. All procedures were performed with closed incision and single pentagon techniques. Outcomes were classified as satisfactory or unsatisfactory, with satisfactory defined as a margin reflex distance of >3 mm and <1 mm between eyelids and unsatisfactory as hypocorrection, surgical complications, and asymmetry.

**Results:**

We included a total of 76 eyelids from 52 patients in our study. Within a mean postoperative follow-up of 16.8 ± 18.5 months (range, 3-95), 59 (77.6%) eyelids had a satisfactory outcome, and 17 (22.4%) were unsatisfactory (8 cases of asymmetry, 3 granulomas, 3 suture extrusions, 2 abscesses, and 1 case of cellulitis). Nine eyelids from the unsatisfactory group required reoperation. Among the patients with a follow-up of ≥12 months (38 surgeries), lasting results were observed in most eyelids, except for 2 late-onset suture extrusions.

**Conclusion:**

The use of polytetrafluoroethylene in frontalis suspension surgery was shown to be predictable, safe, and lasting. Our findings support previous studies that have shown adequate functional results and low complication rates.

## Abstract (pt)

**Objetivo:**

Revisar os resultados de cirurgias de suspensão ao músculo frontal com o uso de fio de politetrafluoretileno em pacientes com blefaroptose.

**Métodos:**

Em um estudo observacional retrospectivo, foram analisados os resultados das cirurgias de músculo frontal de uma instituição, realizadas entre 2003 e 2018. Todos os procedimentos foram realizados com incisão fechada e técnica de pentágono. Os desfechos foram classificados como satisfatórios ou insatisfatórios com definição satisfatória definida como distância margem-reflexo >3mm e <1mm entre as pálpebras e insatisfatória como hipocorreção, complicações cirúrgicas e assimetria.

**Resultados:**

Incluímos um total de 76 pálpebras de 52 pacientes em nosso estudo. Com um tempo médio de seguimento pós-operatório de 16,8 ± 18,5 meses (intervalo 3-95), 59 (77,6%) pálpebras apresentaram desfecho satisfatório e 17 (22,4%) insatisfatórios (8 casos de assimetria, 3 granulomas, 3 extrusões de sutura, 2 abscessos e 1 caso de celulite). Nove pálpebras do grupo insatisfatório necessitaram de reoperação. Entre os pacientes com seguimento ≥12 meses (38 cirurgias), resultados duradouros foram observados na maioria dos casos, exceto por 2 extrusões de sutura de surgimento tardio.

**Conclusão:**

O uso de politetrafluoretileno na cirurgia de músculo frontal mostrou ser previsível, seguro e duradouro, Nossos achados corroboram com estudos prévios que mostraram resultados funcionais adequados e baixos índices de complicação.

## INTRODUCTION

Blepharoptosis is a condition in which the upper eyelid margin is positioned at a lower level than normal in primary gaze^(1)^. The condition can be congenital or acquired and is generally considered congenital if diagnosed during the first year of life. In pediatric patients, blepharoptosis can affect vision and can lead to amblyopia and loss of binocularity^(2)^. Blepharoptosis repair is a challenging problem for oculoplastic surgeons. The choice of surgical procedure depends on the amount of ptosis and levator muscle function. The frontalis suspension (FS) technique is the treatment of choice in severe ptosis with poor levator muscle function (≤4 mm)^(3)^. Various configurations and materials have been described to connect a sling from the upper eyelid to the frontalis muscle, which can be performed using a closed incision or an open incision technique^(4)^.

Autogenous muscle fascia has been widely described as the material of choice in FS surgery; however, this material has been replaced gradually by allogenic material, such as Mersilene, silicone, and polytetrafluoroethylene (PTFE)^(5^,^6)^.

The aim of this study was to review outcomes from our 15 years of experience with FS surgery using PTFE to correct blepharoptosis.

## METHODS

We conducted a retrospective, consecutive, nonrandomized audit of the clinical charts of 57 patients with blepharoptosis who underwent FS with PTFE (model CV3, 6.0-Gore-Tex^®^; W.L. Gore & Associates Inc, Flagstaff, AZ, USA) over a 15-year period (2003-2018) at our ophthalmic outpatient hospital-based clinic in Santo Andre SP, Brazil. All FS procedures were performed using a closed incision and single pentagon technique, as described by Fox^(7)^. The procedures were performed by various sur geons from our teaching hospital. Charts with incomplete examination records and a follow-up of <3 months were excluded. An institutional review board ethics committee approved the study.

We recorded the patients’ epidemiological data, including age, sex, ptosis etiology, surgical outcome, complications, and follow-up time. Surgical outcomes were classified as satisfactory or unsatisfactory. Outcomes with a margin reflex distance (MRD) >3 mm, symmetry (<1 mm MRD difference between eyelids), and no com plications were considered satisfactory. We considered as unsatisfactory outcomes those with remaining asymmetry, hypocorrection, and complications, which included abscess formation, infection, suture extrusion, and granuloma.

## RESULTS

A total of 76 eyelids from 52 patients submitted to FS surgery met the inclusion criteria; 5 patients were excluded according to our exclusion criteria. Twenty-four patients underwent a bilateral procedure. Table 1 shows the epidemiological data. The mean patient age was 17.6 ± 15.6 years (median, 8.0 years; range, 1-55 years), and the mean postoperative follow-up time was 16.8 ± 18.5 months (range, 3-95 months).

**Table 1:** Epidemiological distribution

Category	Number of cases	Percentage
Laterality		
Bilateral	24 patients	46.2%
Right	16 patients	31.0%
Left	12 patients	23.0%
Sex		
Male	38 patients	73.1%
Female	14 patients	26.9%
Etiology		
Congenital	46 patients	88.5%
Blepharophimosis	3 patients	5.8%
Neurogenic	1 patient	1.9%
Trauma	1 patient	1.9%
Marcus-Gunn	1 patient	1.9%
Surgical outcome		
Satisfactory	59 eyelids	77.6%
Unsatisfactory	17 eyelids	22.4%

A total of 59 (77.6%) eyelids had adequate positioning and no complications and were considered as having a satisfactory outcome. Seventeen (22.4%) surgeries were considered as having an unsatisfactory outcome (Table 2): 8 (10.5%) cases of asymmetry or undercorrection, 3 (3.9%) granulomas, 3 (3.9%) suture extrusions, 2 (2.6%) abscesses, and 1 (1.3%) case of cellulitis. In 9 (11.8%) cases, reoperation was required due to abscess (2 eyelids), granuloma (1 eyelid), suture extrusion (3 eyelids), or remaining asymmetry (3 eyelids). The remaining 8 patients did not undergo a second surgical procedure.

**Table 2:** Unsatisfactory postoperative outcomes

Outcome	Reoperation	Number of eyelids	Percentage
Asymmetry	Yes	3	3.9
	No	5	6.5
Granuloma	Yes	2	2.6
	No	1	1.3
Suture extrusion	Yes	3	3.9
Abscesses	Yes	2	2.6
Cellulitis	No	1	1.3

Among the listed complications, 1 case of cellulitis, 1 of granuloma, and 2 of abscesses occurred within the first week of the operation; 2 cases of granuloma and 1 case of suture extrusion occurred within 3 months of the operation; and 1 suture extrusion occurred at 14 months and another occurred at 34 months. Among the cases with a follow-up ≥1 year (38 surgeries with a mean follow-up time of 27.9 months, ranging from 12 to 95 months), there were 2 (5.2%) late-onset suture extrusions that required reoperation. The remaining 36 (94.8%) eyelids maintained their initial characteristics, with no other late-onset complication or significant change in eyelid positioning or symmetry.

## DISCUSSION

Blepharoptosis is a common condition characterized by a low-lying upper eyelid (<2 mm above the middle of the pupil or >2 mm inferior to the upper limbus) in primary gaze. Most cases of blepharoptosis are congenital and unilateral^(8^,^9)^, with the congenital form occurring predominantly due to defective development of the levator muscle (myogenic ptosis), whose fibers present adipose and fibrous tissue, disrupting the proper functioning of the muscle (levator muscle function ≤4 mm). Ptosis can also have neurogenic, mechanical, and aponeurotic causes^(4^,^9)^. Although other studies have shown an equal frequency between sexes^(2)^, our study showed a significant predominance in the males (73%); however, our sample was smaller than those of other studies.

Timely surgical correction of blepharoptosis can prevent irreversible vision loss and provide cosmetic correction^(10^-^12)^. The choice of surgical technique relies on the etiology, extent of ptosis, and levator function. In cases of poor levator muscle function, the standard techniques use the action of the frontalis muscle to raise the eyelids. FS is a minimally invasive technique that connects the frontalis muscle to the eyelid by tissue (muscle fascia) or allogenic material^(2)^ and is typically the technique of choice^(3^,^6)^. Various techniques can be employed to pass the sling from the upper eyelid to the frontalis muscle, including single triangle, single rhomboid (Friedenwald-Guyton), double rhomboid (Iliff), single pentagon (Fox), double triangle (Crawford), and double pentagon^(4)^. The frontalis muscle can likewise be transposed directly to the eyelid, using the frontalis transfer technique^(9)^.

Autologous muscle fascia has been widely employed; however, acquiring the fascia leads to increased surgical times, scar formation in a second surgical site, cicatricial contracture on the upper eyelid, and can hinder possible reoperations. Therefore, allogenic material has been employed increasingly in FS^(5^,^12)^. The use of PTFE for correcting blepharoptosis has been reported over the last 30 years, showing good results with low rates of complication and patient dissatisfaction^(13^,^14)^. Other synthetic materials have been employed also, such as Mersilene and silicone rods^(5^,^6)^. A systematic review has shown that PTFE had the best outcome among the investigated materials^(6)^.

Previous studies^(2^,^6^,^13)^ have shown variable reoperation rates (ranging from 1% to 13%), which correspond to our results (11.8%). We found an infection rate (3.9%) similar to that of previous studies (ranging from 1.7% to 4.3%), and the number of cases in which reoperation was not performed (88.2%) was also similar to frontalis transfer rates (ranging from 81.1% to 87.9%)^(9^,^15)^. The most common complication is undercorrection (ranging from 10% to 15%), although numerous other factors should be assessed, such as eyelid function, corneal protection, diplopia, scarring, and eyelid contour^(2)^.

**Figure 1:**
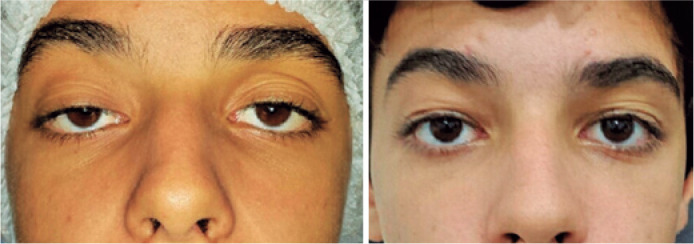
A) Patient with bilateral ptosis B) Late postoperative outcome.

Our study showed satisfactory results (Figure 1), with low overall complication rates. We found that using PTFE was predictable and provided good functional results, with adequate symmetry, eyelid positioning, and contour, along with lasting results in the long-term follow-up. Our study’s findings further support previous studies^(2^,^6^,^13^,^14)^ that consider PTFE an effective and safe material for FS procedures.
